# Human Cytomegalovirus Egress: Overcoming Barriers and Co-Opting Cellular Functions

**DOI:** 10.3390/v14010015

**Published:** 2021-12-22

**Authors:** Veronica Sanchez, William Britt

**Affiliations:** 1 Department of Pediatrics, University of Alabama School of Medicine, Birmingham, AL 35294, USA; wbritt@uabmc.edu; 2 Department of Microbiology, University of Alabama School of Medicine, Birmingham, AL 35294, USA

**Keywords:** human cytomegalovirus, nuclear egress, virus assembly, virus–cell interactions

## Abstract

The assembly of human cytomegalovirus (HCMV) and other herpesviruses includes both nuclear and cytoplasmic phases. During the prolonged replication cycle of HCMV, the cell undergoes remarkable changes in cellular architecture that include marked increases in nuclear size and structure as well as the reorganization of membranes in cytoplasm. Similarly, significant changes occur in cellular metabolism, protein trafficking, and cellular homeostatic functions. These cellular modifications are considered integral in the efficient assembly of infectious progeny in productively infected cells. Nuclear egress of HCMV nucleocapsids is thought to follow a pathway similar to that proposed for other members of the herpesvirus family. During this process, viral nucleocapsids must overcome structural barriers in the nucleus that limit transit and, ultimately, their delivery to the cytoplasm for final assembly of progeny virions. HCMV, similar to other herpesviruses, encodes viral functions that co-opt cellular functions to overcome these barriers and to bridge the bilaminar nuclear membrane. In this brief review, we will highlight some of the mechanisms that define our current understanding of HCMV egress, relying heavily on the current understanding of egress of the more well-studied α-herpesviruses, HSV-1 and PRV.

## 1. Introduction

Herpesviruses follow a complex assembly pathway in which viral DNA is first replicated and encapsidated in the nucleus, followed by the acquisition of a mature virion envelope within cytoplasmic membranes. The nuclear membrane, the protein lattice known as the lamina underlying the nuclear membrane, and chromatin represent significant barriers to the egress of subviral particles from the nucleus. To breach these physical barriers, herpesviruses have evolved mechanisms that include both viral and cellular functions to modify nuclear structures, such as modification of the nuclear lamina that allows access of capsids to the inner nuclear membrane (INM) and facilitates budding into the perinuclear space (PNS). While several components of the viral nuclear egress complex (NEC) that mediate the process of the capsid nuclear exit are conserved between members of the herpesvirus family, cellular functions that contribute to nuclear egress appear to vary among the α, β, and γ subfamilies presumably because of unique host cell responses that follow infection by viruses from each family. Even though different members of the herpesvirus family utilize different cellular functions in transit of their capsids from the nucleus to the cytoplasm, the conservation of the core components of the nuclear egress machinery of these viruses makes the core NEC an attractive target for the development of antiviral drugs to inhibit herpesvirus replication [1].

## 2. Human Cytomegalovirus Replication Cycle

**Figure 1:**
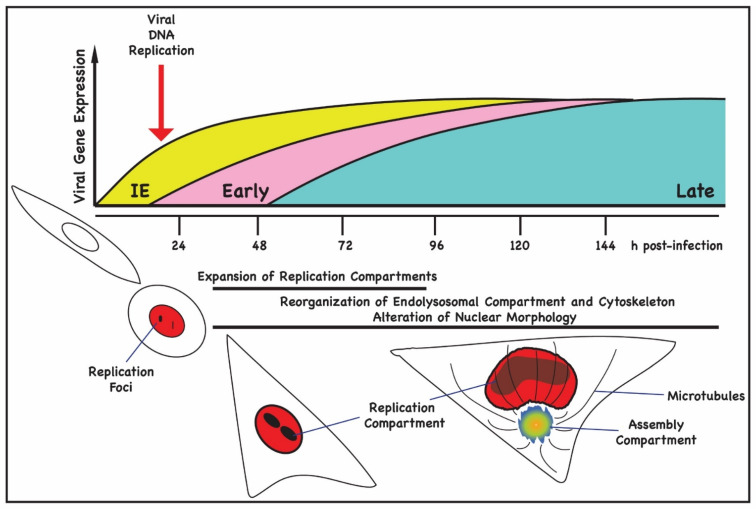
Timeline of viral gene expression and morphological changes in HCMV-infected cells. In the top panel, the kinetics of viral gene is shown. Immediate-early gene (yellow) expression is observed 1 h following infection of permissive human fibroblasts and within 24 h pi, cell rounding is observed, concurrent with the expression of early gene (pink) products. Small replication foci containing IE and E proteins can be detected at this time point. By 48 h pi, cells begin to flatten and enlarge, and nuclear replication compartments can be seen along with expression of late gene (blue) products, including virion structural proteins. In the lower panel, the morphological changes that take place during a permissive infection of human fibroblast cell are illustrated. At 72 h pi, the enlarged nuclei begin to adopt the characteristic kidney shape, and the juxtanuclear assembly compartment (AC) becomes readily detected with microtubules emanating from the Golgi-derived MTOC.

Like other herpesviruses, viral DNA replication occurs in the nucleus, while acquisition of the mature envelope occurs in the cytoplasm, but unlike α-herpesviruses, the HCMV life cycle is protracted and progeny virions are produced over a period of days, as shown in Figure 1. Nonetheless, major steps of the nuclear phase of herpesvirus infections are similar between the different virus families. After entry of HCMV into the host cell, the viral genomes are deposited into the nucleus at PML nuclear domains and circularize. Expression of IE (immediate-early) and E (early) genes is initiated, and viral proteins accumulate at these viral pre-replication sites to stimulate rolling circle replication of the input genomes. As infection proceeds and depending on the multiplicity of infection, cells can contain several replication foci that coalesce into larger replication compartments (RC) containing the replication machinery and early-late proteins required for encapsidation of the viral genome. Concurrent with expansion of the RCs, host DNA is marginalized, and nuclear morphology is altered [2,3,4,5,6] (Figure 1). Although herpesvirus infection-induced changes in nuclear size have been noted by many investigators, advances in microscopy have facilitated accurate measurements of the increases in nuclear volume and overall surface area and of changes in substructures such as the nuclear envelope. For example, Aho et al. measured an average nuclear volume of 260 µm^3^ in HSV-infected cells versus 170 µm^3^ in uninfected controls [5]. The surface area of the nuclei of HSV-infected cells also increased to 260 µm^2^ compared to 176 µm^2^ for uninfected cells [5]. In HCMV-infected cells, nuclei are markedly larger than uninfected cells, and the surface area of the nucleus is further increased by the presence of NE infoldings [4,7,8].

Immediate-early (IE) viral gene products can be detected within 8–12 h post-infection (h pi) in nuclear pre-replication foci after HCMV infection of permissive cells in G_0_/G_1_ [9,10,11] (Figure 1). Viral DNA replication begins by 24 h pi and plateaus by 72–96 h pi (Figure 1). The expression of virion structural genes is an early-late event, and many of the protein products of these viral genes can be detected by 48 h pi. Remodeling of the nuclear lamina and changes in nuclear morphology are detected during a similar time interval after infection. HCMV induces major reorganization of intracellular membranes and dysregulates membrane trafficking to generate a juxtanuclear membrane compartment that is required for efficient cytoplasmic envelopment and virus assembly [6,12,13,14,15]. The morphogenesis of the juxtanuclear site of cytoplasmic envelopment, also known as the assembly compartment (AC), begins at approximately 24 h pi when depolymerization of actin and changes in Golgi morphology are observed [12,13,15,16]. Virus particles exiting the nucleus acquire their mature envelope by budding into AC vesicles that contain virus-encoded glycoproteins and that subsequently fuse with the plasma membrane, releasing virions into the extracellular space. The morphogenesis and function of the AC in virus assembly are areas of intense investigation and beyond the scope of this review; however, there are several structural features of the AC that are relevant to a discussion of nuclear egress [6]. First, the AC serves as a Golgi-nucleated microtubule organizing center [16]. The microtubules that extend from the AC become acetylated, a modification that imparts stability. Acetylation of the microtubules is associated with nuclear rotation, a phenomenon often associated with mechanical stress during cell migration and also during preparation for cell division [16]. Lastly, activity of the AC MTs and their associated motors is required for changes in nuclear morphology observed in HCMV-infected cells [6,16,17]. 

### Overview of Nuclear Egress of HCMV

**Figure 2:**
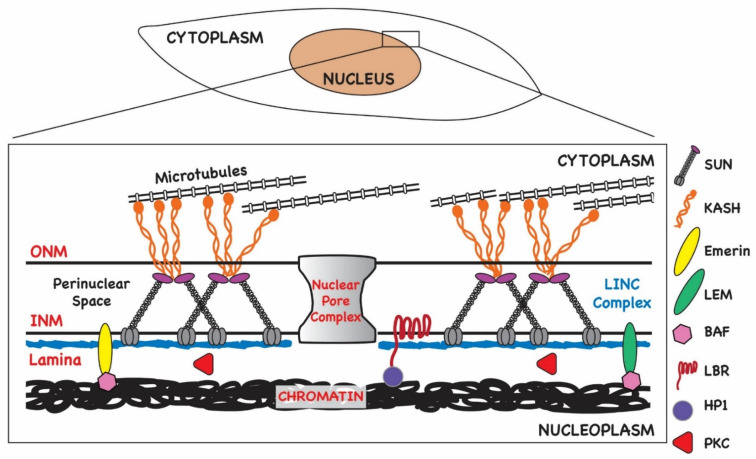
Organization of nuclear envelope in normal cells. The nuclear envelope consists of a double bilayer, the inner nuclear membrane (INM) and outer nuclear membrane (ONM). These membranes are connected at nuclear pores, a protein complex that permits diffusion and active transport of molecules across the envelope. The space between the ONM and INM, the perinuclear space (PNS), is traversed by the LINC (linker of nucleoskeleton and cytoskeleton) complex that is composed of KASH domain proteins (nesprins) anchored in the ONM and SUN proteins anchored in the INM. The lamina underlies the INM and is composed of intermediate filament proteins known as the lamins and is connected to the INM by proteins including the lamin B receptor (LBR) and LEM-domain proteins (Lap2-emerin-Man1). The lamina maintains nuclear shape and stability and serves as a scaffold for other nuclear proteins. BAF (Barrier-to-autointegration factor) binds LEM-domain proteins and chromatin. Phosphorylation of BAF during mitosis leads to its release from chromatin. Similarly, HP1 (heterochromatin protein 1) is a component of heterochromatin that links condensed chromatin to the INM through LBR.

Following viral DNA replication and packaging of newly formed capsids, genome-containing capsids must transit from RC and cross the nuclear envelope through a process of envelopment at the inner nuclear membrane (INM) and de-envelopment at the outer nuclear membrane (ONM) to reach sites of tegumentation and final envelopment in the cytoplasm (Figure 2). In addition to the nuclear membrane, several other barriers restrict the egress of capsids from the viral replication centers to the cytoplasmic AC, including the nuclear lamina and chromatin (Figure 2). Modification of these nuclear structures during herpesvirus egress are briefly reviewed below. 

## 3. Interphase Nuclear Architecture

The nuclear envelope consists of a double bilayer, the INM and the ONM, as well as the underlying lamina (Figure 2). The ONM is contiguous with the endoplasmic reticulum (ER), thus allowing lateral diffusion of membrane-bound proteins destined for localization within nuclear membranes. In addition, since the ER is the main site of phospholipid synthesis, the continuity of the ONM with the ER allows for nuclear membrane expansion by lateral diffusion of lipids, although local synthesis and storage of lipids at the INM have been described [18]. In fact, local synthesis of phospholipids has been shown to contribute to the formation of NM invaginations known as the nucleoplasmic reticulum (NR) [19]. One potential function proposed for the NR is to facilitate communication from the nuclear periphery to inner domains of the nucleus. Local phospholipid accumulation in the INM has also been shown to be regulated by host cell functions contained within the endosomal sorting complex required for transport III (ESCRT-III), a complex of proteins that has a critical role in vesicle formation, abscission during cytokinesis, and the budding of enveloped viruses [20,21]. Proteins within this complex have been shown to contribute to the regulation of INM proliferation and repair during mitosis, resealing nuclear ruptures in migrating cells, and, most recently, nuclear egress of herpesvirus particles [20,21,22,23]. 

Nuclear permeability is maintained by nuclear pores that serve as junctions between the INM and ONM (Figure 2). Pore complexes are large, multi-subunit structures 110 MDa in size (~800 Å wide) that allow nucleocytoplasmic transit of macromolecules such as RNAs and proteins in an energy-dependent manner [24]. Smaller molecules (<40 kDa) can diffuse through pore channels without ATP consumption. The intermembrane or perinuclear space (PNS) between the INM and ONM spans approximately 20–40 nM and contains components of the LINC (linker of nucleoskeleton and cytoskeleton) complex (Figure 2). LINC complexes function as mechanosensors relaying signals to and from the nuclear environment and cytoplasm [25]. They are composed of a trimer of SUN-domain (Sad1 and UNC-84) proteins anchored in the INM that are in turn bound to a trimer of nesprins (KASH domain proteins) anchored in the ONM. Thus, the LINC complex extends from the INM and PNS into the cytoplasm (Figure 2). Nesprins bind to cytoskeletal elements such as tubulin, and thus the LINC complex provides a bridge to the karyoskeleton both structurally and for intracellular signaling. For example, the LINC complex has been implicated in the regulation of transcription in response to changes in the extracellular matrix [26]. The LINC complex is also important for nuclear positioning [27,28,29,30]. 

Together, the lamina, the nuclear pores, the LINC complex, spectrin, actin, and the LEM-domain proteins, which are involved in tethering chromatin to the NE, all contribute to maintaining nuclear architecture in interphase cells [31,32] (Figure 2). Regulation of components of the nucleoskeleton effect changes in nuclear structure and function during the cell cycle, during stress responses, and in response to extracellular cues that trigger cell migration. The dynamic nature of nuclear architecture is essential for homeostasis and genome maintenance. Like other viruses that replicate in the nucleus, herpesviruses take advantage of the plasticity of nuclear structure to create an environment that favors the replication of the viral genome over that of the host without compromising the integrity of the nuclear compartment prematurely in order to maximize virus production. 

## 4. Herpesviruses Circumvent Nuclear Barriers 

Perhaps the most well understood step in the nuclear egress of α, β, and γ herpesviruses is the transit of the viral capsid through the INM into the perinuclear space (PNS) and in particular, the role of viral proteins in this step of the assembly pathway. Budding of membrane-bound capsids from the INM into the PNS, alternatively termed primary envelopment, has been most been well studied in the α-herpesviruses herpes simplex virus (HSV) and pseudorabies virus (PRV). Primary envelopment of α-herpesviruses is dependent on the interaction between a heterodimer of pUL31, a nuclear phosphoprotein, and pUL34, a type II membrane protein inserted into the INM, both of which are required for budding of the capsid into the PNS [33,34]. Together these viral proteins constitute the nuclear egress complex (NEC). Interactions between these core NEC proteins and formation of the heterodimer have been argued to lead to the recruitment of other viral proteins, including viral kinases and host cell proteins that are required for efficient passage through the INM. The NEC is conserved in all families of herpesviruses. In the prototypic β-herpesvirus, HCMV, the NEC consists of the pUL53 and pUL50, orthologs of the HSV pUL31 and pUL34 respectively, and a heterodimer of HCMV pUL53 and pUL50 has also been shown to contribute to the recruitment of viral and cellular proteins that contribute to nuclear egress [35,36]. Similarly, the NEC of the γ-herpesvirus EBV has been identified and is represented by the HSV pUL31 and pUL34 orthologs, BFLF2 and BFRF1, respectively [37,38,39]. A further description of the function of the HCMV NEC will be provided in greater detail in the following sections.

In the case of PRV, the interaction between the orthologs of HSV pUL31 and pUL34 is sufficient for vesiculation of the INM and formation of particles resembling L particles of α-herpesviruses in transfected cells in the absence of expression of other PRV encoded proteins, arguing that interactions between the NEC and the PRV capsid are not required for budding of particles at the INM [40]. Furthermore, purified pUL31 and pUL34 of HSV and the PRV orthologues can vesiculate membranes in cell-free systems, strongly suggesting that the minimal NEC of α-herpesviruses is sufficient for budding from lipid-containing membranes [40,41,42,43]. Although these observations could be interpreted as evidence that other viral and host cell functions are non-essential for herpesvirus nuclear egress, multiple studies have shown that efficient nuclear egress leading to WT levels of virus replication requires additional viral proteins together with the viral NEC. Furthermore, it could be argued that cellular proteins can functionally complement or even partially replace viral functions encoded by non-NEC viral proteins. Consistent with this possibility is the finding that although the deletion of the PRV-encoded protein kinase pUS3 led to large invaginations of the INM and to an increased number of primary enveloped capsids in the PNS in PRV-infected cells, this deletion had a marginal effect on the production of infectious virus [34,44], suggesting that other redundant functions compensate for the loss of US3. Consistent with this possibility, protein kinase C (PKC) was argued to disrupt the nuclear lamina during nuclear egress of murine CMV, whereas more recent findings have argued for a more prominent role of the conserved herpesvirus protein kinase (CHPK) of HCMV, pUL97, in this step in nuclear egress [45,46]. A number of other host cell functions have been proposed to contribute to budding of the herpesvirus capsid at the INM, including emerin and p53, and deletion or decreased expression of these cellular functions in some cases has been shown to result in a quantifiable change in nuclear egress and the production of infectious virus (Table 1) [35,47,48]. Thus, current data suggest that herpesviruses have usurped physiologic processes to facilitate efficient nuclear egress and, ultimately, virus replication. In the following sections, we will attempt to update the current understanding of the role of cellular and viral proteins in HCMV nuclear egress, often in the context of the more well-studied and well-understood models of the nuclear egress of α-herpesviruses. 

### 4.1. Nuclear Egress: Budding of Newly Assembled HCMV Capsids into the PNS

#### Role of Virus-Encoded Proteins

Several detailed descriptions of the transit of the α-herpesvirus capsid through the INM have been published, and because studies have shown that HCMV follows a similar overall pathway for budding into the PNS, only a brief description of this process will be provided with features unique to the transit of HCMV into the PNS highlighted. As noted previously, the HCMV-encoded proteins of pUL50 and pUL53 are the core components of the HCMV NEC, and deletion of either viral gene is lethal; thus, both are classified as essential viral genes [72]. Neither viral protein has been convincingly detected in the mature extracellular virion by mass spectrometry; however, early studies using immunoelectron microscopy suggested that cytoplasmic-enveloped particles contained pUL53 [49]. pUL53 is a 367 aa nuclear phosphoprotein with a defined monopartite nuclear localization signal (NLS) that results in its localization in the nucleus in the absence of other viral proteins [50]. pUL53 has been shown to interact with pUL50 as well as a number of other proteins, including pUL97 [35,46,57,60]. pUL50 is a 397 aa type II membrane protein anchored in the INM with the C-terminal 16 aa in the PNS [61]. Of note, HCMV pUL50 is approximately 125 aa larger than the HSV-1 ortholog and 50 aa larger than the EBV ortholog. pUL50 does not express an identified NLS and is thought to localize to the INM following insertion into the ER membranes and translocation through the membranes bridged by nuclear pores that are contiguous with the INM [50]. Expression of pUL50 in the absence of other viral proteins resulted in its localization in the INM [50]. Deletion of the transmembrane domain of pUL50 redirected its expression to the cytoplasm, and expression of this pUL50 mutant with pUL53 results in its localization in the nucleus, not the INM, consistent with the requirement of the TM domain for its INM localization [46,50,73]. Both biochemical and structural studies have described in considerable detail the interaction between pUL50 and pUL53 that leads to the formation of a heterodimer that constitutes the NEC of HCMV [51,52]. Structural studies of the NEC from both α- and β-herpesviruses have described a heterodimer with a hook-into-groove type interaction [43,51,52,53,54,74,75]. The groove component of this heterodimer encoded by pUL50 consists of four helical segments and an anti-parallel β-sheet sandwich [51,53]. The hook component encoded by pUL53 contains two consecutive α-helices within a short strand of residues (aa 59–87) in the N-terminus [51,53]. Although this structure is highly conserved for all NEC thus far described, it is interesting that the primary sequence of the core NEC proteins is not highly conserved between different herpesviruses, including contact residues within the hook-into-groove interfaces [75]. 

Structures of the NEC complex of HSV derived by cryoelectron microscopy revealed that the pUL31/pUL34 hetrodimer forms a hexameric ring-like structure in the presence of membranes [41]. The oligomerization of the pUL31/pUL34 heterodimer into the hexameric ring is required for egress as mutations that disrupt the oligomerization also block egress [41,76,77,78]. A similar ring-like structure of the heterodimer of HCMV pUL50/pUL53 has also been described for the HCMV NEC [51]. This ring-like structure consists of a pUL50 hexamer with pUL53 located between individual pUL50 molecules, thus contacting two pUL50 molecules but not in contact with adjacent pUL53 molecules [51]. The hexameric structure of the NEC appears highly conserved in herpesviruses, thus suggesting a common function of this structure in this large family of viruses possibly acting as a scaffold for the association and/or recruitment of other viral and cellular proteins that could facilitate the budding process. Although the hexameric ring-like structure of the NEC heterodimers has been confirmed using both cell-free systems and infected cells, the planar structure of the hexameric ring has also presented a conundrum for mechanisms of budding at the INM, a process that includes membrane deformation resulting in the generation of spherical enveloped capsids in the PNS [42]. Recently, this question was further addressed in a study of nuclear egress of HSV using cryoelectron microscopy. These investigators demonstrated that HSV pUL25 interacts with membrane-associated pUL31, leading to the formation of NEC pentagons that are proposed to secure the NEC to the vertices of the capsid [42]. Based on these findings, these authors have proposed that irregular incorporation of pUL25 containing NEC pentagons into the hexameric NEC lattice can disrupt the hexameric ring, thus permitting the formation of an icosahedral-like structure surrounding the capsid and the budding of a spherical structure into the PNS [42]. 

The interaction between pUL50 and pUL53 has been shown to result in concentration of the heterodimer in the INM, a key step in nuclear egress [50]. In addition, more recent findings have argued that the interaction between pUL50 and pUL53 could also facilitate the recruitment of other viral and cellular proteins that contribute to nuclear egress to the NEC [79]. Several host proteins were identified using co-immune precipitation protocols, and the analysis of the NEC by mass spectrometry identified additional viral and host proteins associated with the NEC [35]. The CHPK of HCMV, pUL97, is the most consistently detected viral protein associated with the NEC of HCMV and is thought to interact specifically with pUL53. As discussed in more detail in the following sections, pUL97 has been shown to have a critical role in egress secondary to its role in phosphorylation of the nuclear lamina leading to disruption of this barrier [46,57,62]. 

More recent studies have identified a number of HCMV capsid components associated with pUL53 in co-immunoprecipitation assays [79]. Furthermore, the association of pUL53 with intranuclear capsids was confirmed using immunoelectron microscopy [79]. These observations led these investigators to propose that intact capsids could interact with pUL53 prior to the interaction between pUL53 and pUL50, thus providing a central role of UL53 in the recruitment of capsids to budding sites on the INM [79]. The interactions between pUL53 and intranuclear capsids is thought to require a viral adaptor protein(s), a postulate based on studies of the role in egress of the α-herpesviruses capsid-associated proteins, pUL17 and pUL25 [80,81,82,83]. Findings from studies in PRV and HSV have proposed that the pUL25/pUL17 heterodimer serves as an adaptor between capsids and the NEC, thus facilitating targeting of capsids and subsequent budding from the INM [82,84]. More recently, specific residues on pUL31 required for capsid binding to the NEC have been identified, suggesting the likelihood of interactions between pUL25, the capsid, and the NEC [85]. Mutations of these residues in pUL31 resulted in mutant viruses with reduced virus yield, increased numbers of capsids in the nucleus, and empty vesicles in the PNS [85]. However, it should be noted that other roles in assembly have been proposed for these α-herpesvirus proteins, including a role of encapsidation for PRV pUL17 [83]. Interestingly, pUL25 of HSV has been reported to be required for genome encapsidation, whereas the PRV pUL25 is not required for the encapsidation of newly replicated viral DNA [40,86].

Candidates for the function of a viral adaptor protein linking the capsid to the NEC in HCMV include pUL77 (HSV UL25 ortholog) and pUL93 (HSV UL17 ortholog). Both proteins are expressed with early late kinetics and interact to form a pUL77/pUL93 heterodimer [55,56]. Imaging studies have indicated that both proteins were localized with capsid proteins and, perhaps, intranuclear capsids [55]. Co-immunoprecipitation assays demonstrated interactions between pUL77 and capsid proteins MCP (pUL86), SCP (pUL49a), mCP (pUL85), and mCP-BP (pUL46) [55]. In contrast to reports that described preferential binding of the α-herpesviruses pUL25/pUL17 heterodimer to intranuclear C capsids as a mechanism to enrich primary envelopment of infectious capsids during nuclear egress, neither HCMV pUL77 nor pUL93 exhibited preferential binding for intranuclear A, B, or C capsids [55,87,88]. Nonetheless, both pUL77 and pUL93 were tightly associated with capsids in extracellular virions [55]. Unit length genomic DNA could not be detected in cells infected with mutant viruses in which either UL77 or UL93 was deleted, suggesting that these proteins were required for viral DNA encapsidation [55]. 

Lastly, virion proteins could directly regulate the function of components of the NEC, thus providing another level of regulation of egress. As noted previously, deletions of the α-herpesviruses protein kinase, pUS3, have been shown to alter the capsid egress with the resulting accumulation of NEC-coated particles in the PNS; however, whether similar phenotypes would be observed following mutation of proteins that regulate pUS3 function such as has been suggested for the CHPK of α-herpesviruses, UL13, is less well understood [89,90,91,92,93,94]. Although HSV pUL13 has been shown to phosphorylate HSV pUS3 in vivo and in vitro, the importance of this pUL13-mediated post-translational modification of pUS3 in phosphorylation of components of the NEC of HSV is unclear [94]. More recently, pUL21 of HSV has been shown to play a role in the regulation of phosphorylation of pUS3 as well as proteins of the NEC, and it has been shown that the phenotype of pUL21 deletion mutant viruses could be secondary to the hyperphosphorylation of components of the NEC and defective primary envelopment at the INM [95]. In the case of HCMV, pUL97 has been shown to phosphorylate both pUL50 and pUL53 and to have autophosphorylating activity [58,59]. However, it remains unknown if other HCMV proteins also regulate the functions of pUL97 required for efficient nuclear egress.

### 4.2. Role of Cellular Proteins in Nuclear Egress of HCMV: Modification of Cellular Barriers to Capsid Budding into the PNS

#### 4.2.1. Chromatin as a Barrier to Nuclear Egress

In the context of viruses that replicate in the nucleus of interphase cells, cellular chromatin represents a significant barrier to the virus in terms of competing for the availability of nucleotides and other factors required for replication and egress of subviral particles from the nucleus. The replication of herpesviruses causes marginalization of the cellular chromatin towards the nuclear periphery as RC expand [2,3,5,8]. Importantly, nascent capsids must cross this barrier to access the INM. 

**Figure 3:**
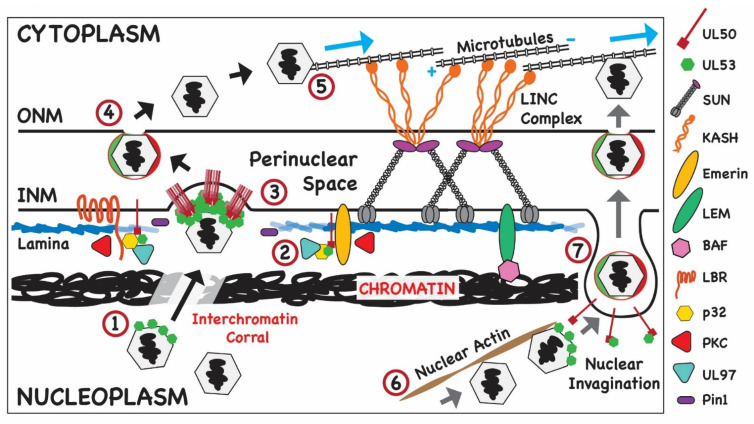
Modification of nuclear structures that allow HCMV nuclear egress. The structure of nuclear membrane described in Figure 2 is shown with modification of nuclear structure that could act as barriers for egress of HCMV capsids. (1) Newly formed capsids decorated with pUL53 are shown transiting chromatin through an interchromatin corral; (2) disruption of the nuclear lamina by host cell proteins such as Pin 1 and the viral protein kinase, UL97; (3) loss of this barrier followed by heterodimerization of the core NEC of HCMV (pUL53 and pUL50) at the INM promotes primary envelopment of capsids at the INM. (4) Following primary envelopment at the INM and transit through the PNS, the enveloped capsid then fuses with the ONM and undergoes deenvelopment, releasing the particle into the cytoplasm; (5) following acquisition of the inner tegument proteins, the tegumented capsid is transported on MT to sites of cytoplasmic assembly. (6) Alternatively, the capsid could be transported by nuclear actin to sites of budding from the INM; (7) following interactions between accessory viral proteins and pUL53 of the NEC, heterodimerization of pUL53 and pUL50 leading to primary envelopment at a nuclear invagination generated through disruption of the nuclear lamina as described above. Following primary envelopment, transit through the PNS space and fusion with the ONM, the capsid is released from ONM and trafficked to sites of virus assembly in the cytoplasm as described above.

A cellular protein that has been reported to be involved in nuclear egress and potentially in the regulation of INM infoldings is WDR5 (WD repeat domain 5 or WD repeat-containing protein 5) [65]. This nuclear protein is highly conserved and is a member of SET1/MLL1 complexes that methylate histone H3, thus regulating chromatin structure and accessibility to the transcriptional machinery [96,97,98]. Like the nuclear lamina, chromatin is a recognized factor in nuclear plasticity, and WDR5 has been implicated in the modulation of nuclear morphology related to chromatin structure independent of its role in transcription [98]. H3K4 methylation by WDR5-containing complexes causes chromatin decompaction, making the nucleus less rigid and thereby facilitating nuclear deformation in response to environmental cues [97,98]. Based on these and other observations, it could be speculated that a linkage between epigenetic marks and nuclear deformation is required for optimal nuclear egress of herpesviruses. In the context of HCMV infection, WDR5 has been implicated in primary and secondary envelopment [65]. In cells depleted of WRD5 by siRNA, the distribution of the NEC on the rim of the INM in infected cells was heterogenous in comparison to control cells with a reduced number of pUL53-positive nuclear puncta and fewer cytoplasmic virions compared to control cells [65]. Increased expression of WDR5 during infection could promote decompaction of cellular chromatin as it is pushed to the nuclear periphery by the expansion of the viral replication compartment, thereby reducing the nuclear barrier imposed by chromatin and permitting movement of capsid to sites of budding on the INM [65,99,100,101]. Studies by Procter et al. described the polarized accumulation of condensed histone H3K9me3 host DNA at a site proximal to the AC, while active H3K4me3-marked DNA appeared to be distributed uniformly across the nucleus relative to the AC [8]. These data suggest that the area of the nucleus immediately adjacent to the AC is rigid as a result of the underlying chromatin structure, while the remainder of the nuclear membrane is more flexible as a result of chromatin decompaction. From this, it is inferred that the nuclear membrane closest to the AC would represent a significant barrier to capsid egress, as exit would be inhibited by condensed chromatin. However, it should be noted that Buchkovich et al. demonstrated that the area of the nucleus proximal to the AC was permeable to high molecular weight dextran at late times post-infection, consistent with their observations from EM studies that the ONM was not intact in that location [4]. These authors speculated that the increased permeability at this area of the nucleus would accelerate nuclear egress and provide a mechanism for vectorial transport of capsids to the AC. One way to reconcile these data is to propose that the accumulation of condensed histone H3K9me3-marked chromatin helps to maintain the integrity of the NE next to the AC that is otherwise compromised by the reorganization of cellular membranes and the cytoskeleton during infection. Lastly, data from α-herpesvirus infected cells support a model in which capsids diffuse to the INM through channels or corrals in the chromatin [5,102] (Figure 3). Thus, it is possible that the formation of such channels in the marginalized cellular DNA also occurs in HCMV-infected cells, including the chromatin located closest to the AC. Capsid diffusion may occur in tandem with more directed types for trafficking mediated by nuclear actin and myosin Va, which is discussed below [66,67].

#### 4.2.2. Modifications of Nuclear Lamins and HCMV Nuclear Egress

The plasticity of the nucleus is controlled in large part by lamin polymerization. In mammalian cells, there are three lamin genes encoding lamins A and C, through alternative splicing, and lamins B1 and B2, which are distinct proteins that share about 60% identity [103]. All lamins share a similar structure consisting of a central rod domain flanked by an N-terminal head and C-terminal tail. In A- and B-type lamins but not lamin C, the tail contains a CaaX-motif that is the site of farnesylation and extensive processing, which results in membrane targeting. All domains of the lamins are required for polymerization that involves the head-to-tail polymers of dimers and subsequent assembly into protofilaments and intermediate filaments. Importantly, lamins are targets for several types of post-translational modifications including phosphorylation, acetylation, addition of lipid moieties (farnesylation and myristoylation), and proteolytic cleavage. These modifications, particularly phosphorylation, regulate polymerization of the lamina and protein–protein, as well as protein–DNA, interactions [104]. Several kinases target the lamins including protein kinases A and C, cyclin-dependent kinase 1 (Cdk1), and mitogen-activated protein kinase (MAPK), and because phosphorylation is reversible, this modification plays a key role in controlling the structure and function of the nuclear lamina; PP1 and PP2A mediate the dephosphorylation of lamins upon mitotic exit, allowing for lamin polymerization after cell division [103].

As discussed earlier, studies of the mechanism of MCMV capsid egress suggested that host proteins played a critical role in remodeling the nuclear membrane during nuclear egress (Figure 3). One such observation detailed the phosphorylation of the nuclear lamina by the protein kinase C (PKC) during nuclear egress of MCMV [45]. Subsequent studies provided compelling data that the CHPK of HCMV, pUL97, is responsible for the phosphorylation of lamins A/C and that this viral protein kinase mimics the activity of the host cell mitotic kinase, Cdk1 [46,57]. Consistent with this interpretation is the finding that UL97 phosphorylates Ser22 of lamin A/C, which is also a Cdk1 consensus site [57]. Although protein kinase C has been implicated in disruption of the lamina in cells infected with MCMV, Sharma et al. could not demonstrate recruitment of PKC to the lamina in cells infected with HCMV [46]. Moreover, a pan-PKC inhibitor did not block dissolution of the lamina in HCMV-infected cells [105]. More recent studies have described additional cellular proteins that are associated with the NEC and/or proposed to have a role in HCMV nuclear egress [35,36,65] (Table 1). For example, Pin 1 has been proposed to play a role in the disruption of the lamina in HCMV-infected cells [63]. Phosphorylation of lamins at consensus Cdk1 S/TP sites recruits isomerases that catalyze the conversion of proline from the *cis* to *trans* conformation, a modification that introduces kinks into the secondary structure of lamin that can then alter protein interactions and lamin polymerization [104]. Pin1 levels have been reported to increase with early kinetics during HCMV infection [63]. In addition, Pin1 has been shown to co-precipitate with lamin A in lysates from infected cells and localize to the nuclear envelope late in infection in association with the HCMV CHPK, pUL97 [63] (Figure 3). In the absence of Pin1, disruption of the nuclear lamina by pUL97 is decreased, suggesting that Pin1 participates in the modification of the nuclear lamina during HCMV infection [64]. 

#### 4.2.3. Nuclear Actin and Nuclear Egress

Along with microtubules, actin filaments are considered major components of the cytoskeleton; however, nuclear actin, which represents about 20% of the cellular pool of actin, is less well characterized than its cytoplasmic counterpart. In the nucleus, actin has multiple roles including regulation of chromatin, transcription, and movement of chromosomes. The role of nuclear actin in herpesvirus-infected cells remains unresolved, but data suggest that nuclear actin may contribute to nuclear expansion and capsid motility in a virus- and cell-type dependent manner.

A role for nuclear actin in the herpesvirus replication cycle was first suggested by Forest et al. who reported that intranuclear trafficking of capsids was temperature- and energy-dependent, as well as sensitive to an inhibitor of actin polymerization, latrunculin A (LatA) [106]. Capsid movement was also reduced in the presence of 2,3-butanedione monoxime (BDM), a putative inhibitor of the actin-associated molecular motors, myosins, further suggesting an important role for actin in intranuclear capsid trafficking, although the mechanism(s) of action of BDM remains unclear [106]. Interestingly, capsid movement was not affected by another inhibitor of actin polymerization, cytochalasin D (CytoD), which has a mode of action distinct from LatA, which was attributed at the time to differences between the nuclear and cytoplasmic actin networks [106]. Later work by Simpson-Holley et al. demonstrated that the treatment of HSV-1 infected cells with LatA but not CytoD led to significant alteration in nuclear size and distribution and expansion of viral replication compartments relative to control infected cells, suggesting that nuclear actin acts as a scaffold supporting the changes in nuclear architecture observed during infection [107]. Importantly, these authors reported that even though LatA inhibited nuclear expansion during infection, focal disruption of the lamina caused by infection was still observed in the presence of the actin polymerization inhibitor; moreover, LatA treatment did not reduce virus yields at the concentrations used [107]. 

Both of the studies described above suggested that nuclear actin plays a role during the herpesvirus life cycle in mediating nuclear expansion and capsid transport; however, these assertions have been challenged by results from other studies. Feierbach et al. were the first to report induction of nuclear actin filaments in neurons infected with the α-herpesviruses PRV and HSV-1 [108]. In addition, these authors reported the association of capsids with nuclear actin filaments, which were found to be required for formation of capsid assembly domains termed assemblons [108]. Capsids were also found to colocalize with myosin V, suggesting a role for actin filaments in capsid transport [108]. Later work by Bosse et al. contradicted this hypothesis by demonstrating that intranuclear capsid trafficking of HSV-1, murine cytomegalovirus, and MHV68 capsids occurs in primary murine fibroblasts (MEFs) that do not contain discernable nuclear actin filaments after infection [109]. Moreover, inhibitors of actin polymerization, including LatA, did not affect intranuclear capsid motility in murine fibroblasts [109]. Thus, the authors concluded that intranuclear capsid motility is not dependent on nuclear actin in MEFs and other cells in which nuclear actin filaments are not induced by infection [109]. In a subsequent publication, these authors suggested that capsid motility occurs by diffusion [102]. 

In contrast to these studies, Wilkie et al. demonstrated a role for nuclear actin in egress in the context of HCMV infection [66] (Figure 3). These authors demonstrated that nuclear actin filaments were induced in cells infected with HCMV AD169 by 6 h p.i. and that their formation required IE gene expression [66]. HSV-1 did not induce nuclear actin in the same cells, suggesting that induction of nuclear actin filaments is cell type- and herpesvirus-specific. The accumulation of nuclear actin filaments continued until late times post infection in HCMV-infected cells, and the filaments were shown to extend from the viral RC to the nuclear rim. These observations suggested that the actin filaments could play a role in the trafficking of nascent capsids to the NEC. Depolymerization of nuclear actin by treatment of cells with LatA at late times post-infection reduced the number of capsids in the cytoplasm and decreased viral titer, again suggesting that nuclear actin plays a role in nuclear egress [66]. Importantly, the concentrations of LatA that were used in these studies to alter virus assembly were also shown to lead to depolymerization of nuclear actin [66]. Electron microscopy revealed that in the absence of nuclear actin, capsids were more often associated with the RC, and fewer were observed near the nuclear rim [66]. Taken together, these data suggested that nuclear actin could play a significant role in the movement of capsids from the RC to the NE (Figure 3). This model was supported the finding that myosin Va, a molecular motor associated with nuclear actin, was also required for nuclear egress [67]. Importantly, these authors showed colocalization between capsids and myosin Va by EM and of the major capsid protein, myosin Va, and nuclear actin filaments by immunofluorescence [67]. Similar to the findings that were obtained when inhibitors of nuclear actin polymerization were used, inhibition of myosin Va activity with siRNA or expression of a dominant negative allele decreased the production of infectious virus, the accumulation of cytoplasmic capsids, and the trafficking for capsids from the RC to the NE [67]. These results strongly implicate nuclear actin and myosin Va in nuclear trafficking and egress of HCMV capsids in infected human fibroblasts. As mentioned above, although nuclear actin has been shown to be important in RC distribution and expansion in HCMV- and HSV-1-infected cells [107], the data from other studies of this herpesvirus support a model in which capsids diffuse to the NE rather than using nuclear actin in their trafficking itinerary within the nucleus [5,102]. 

#### 4.2.4. ESCRT-III and Nuclear Egress

Although several studies have reported that overexpression or inhibition of various cellular functions resulted in decreased virus replication, the impact on virus yield was often less than an order of magnitude, suggesting redundancies in the contribution of individual host cell functions to nuclear egress of herpesviruses. In contrast to these findings, a striking phenotype in the nuclear egress of HSV has been described in infected cells following knockdowns of members of the endosomal sorting complex required for transport (ESCRT) [23]. This well-studied cellular complex plays a role in a wide spectrum of cytoplasmic membrane remodeling and membrane scission events. Relevant to this review, ESCRT-III has also been shown to contribute to budding processes from the nuclear envelope, including repair of the nuclear membrane during cytokinesis and maintenance of the nuclear membrane integrity in interphase cells [20,22,110]. Because components of the ESCRT-III system play a critical role in maintaining the structure and function of the INM, it is not surprising that INM deformation and rupture during herpesvirus egress likely would elicit this cellular response [111,112]. Previously, the ESCRT-III pathway has been shown to play a role in EBV nuclear egress [37]. This study suggested that recruitment of an adaptor of ESCRT-III, ALIX, by the pUL34 EBV ortholog BFRF1 to the INM rim was required for efficient EBV egress [37]. More recent studies have shown that depletion of ALIX reduced recruitment of the HSV NEC to the INM and decreased virus yield; however, the latter finding could also be consistent with the role of ESCRT-III in cytoplasmic envelopment [23]. Importantly, this same report described an increase in primary enveloped capsids in the nucleoplasm and the accumulation of partially enveloped capsids in the PNS as compared to control cells, strongly suggesting a role for ESCRT-III in the nuclear egress of HSV-1 [23]. Deletion of a component of ESCRT-III, the charged multivesicular body protein 4B (CHMP4B), in HeLa cells followed by siRNA knockdowns of CHMP4A and CHMP4C and infection with HSV-1 resulted in a similar phenotype as deletion of the ESCRT-III adaptor ALIX [23]. In this latter experiment, the yield of infectious virus fell by about 3 log_10_, and consistent with the initial observation, there was an accumulation of enveloped capsids associated with INM invaginations in the nucleoplasm, suggesting a failure of capsids to successfully transit through the INM [23]. Consistent with these findings, a similar phenotype was described when a dominant negative VPS4 ATPase was used to alter ESCRT-III function in HSV infected cells [23]. Lastly, a recent study from the same group of investigators provided additional evidence for the role of the ESCRT-III complex in nuclear egress by demonstrating that infection with a replication competent HSV-1 containing a mutation in the disordered domain of pUL34 led to the altered localization of the NEC-infected cells, loss of pUL34 colocalization with CHMP4B of the ESCRT-III complex, and the accumulation of enveloped capsids in the PNS [68]. In contrast, studies in HCMV have thus far failed to provide convincing evidence for a role of the ESCRT-III pathway in nuclear egress, although it is important to note that studies to specifically determine the role of the ESCRT-III in HCMV nuclear egress have not been accomplished presumably because of the experimental challenge to deconvolute the impact of ESCRT-III functions on nuclear and cytoplasmic phases of virus assembly. For example, in one study, inhibition of ESCRT-III function by overexpression of a dominant negative VSP4a inhibited virus production, presumably through blocking cytoplasmic envelopment of HCMV [113]. However, definitive evidence excluding the potential role of the expression of the dominant negative VPS4 on nuclear egress was not explored [113]. A more definitive series of experiments using dominant negative constructs to inhibit ESCRT-III function that also controlled for the effects of overexpression of these dominant negative proteins on cell viability failed to demonstrate a significant impact on envelopment and the release of infectious HCMV, a finding, as noted above, that was inconsistent with results from similar experiments in studies of HSV-1 [23,114,115]. Although the results from various studies of the role of the ESCRT-III complex in nuclear egress appear contradictory, the experimental approaches used in these studies vary considerably, ranging from dominant negative mutants, siRNA depletions, CRISPR-derived knockout cells, and recombinant viruses. Yet, a summation of the data from studies in α and γ herpesviruses strongly suggests a role of ESCRT-III in nuclear egress and likely illustrates another example of herpesviruses co-opting a normal cellular function for more efficient replication. 

### 4.3. Nuclear Egress: Transit of Newly Assembled HCMV Capsids from the PNS through the ONM to the Cytoplasm

#### 4.3.1. Role of Viral Proteins

Although an extensive amount of literature describing the primary envelopment of herpesvirus capsids at the INM and movement into PNS is available, the transit of the enveloped capsid through the PNS and ONM to the cytoplasm of the infected cells, including that of HCMV, remains understudied and largely undefined. As discussed previously, the deletion of the α-herpesvirus protein kinase, pUS3, has been reported to result in the accumulation of enveloped capsids in the PNS [44,89]. Deletion of HSV gB and gH led to a significant defect in nuclear egress that was characterized by accumulation of enveloped particles in the PNS and herniations of the INM, suggesting that deletion of these viral glycoproteins that constitute the fusion machinery of herpesviruses resulted in the failure of enveloped capsids to fuse with the ONM [116]. Subsequently, a similar nuclear egress phenotype was observed in cells infected with HSV mutant virus in which only the fusion loops of gB were mutated [69]. These findings strongly argued that HSV gB, which has been shown to require expression of gH/gL for the fusion of incoming virions in permissive cells, was required for fusion at the ONM of primary enveloped capsids during nuclear egress. However, similar phenotypes have not been observed in PRV mutant viruses with deletions of homologous envelope glycoproteins [117]. To date, the differences between the nuclear egress phenotypes of these two closely related α-herpesviruses following deletion of essential glycoproteins have not been resolved. In the case of HCMV, there are no conclusive data relevant to its transit through the PNS and ONM to the cytoplasm. 

#### 4.3.2. Role of Cellular Proteins

During formation of the nuclear pores complex (NPC) in interphase cells, fusion between the INM and ONM has been proposed to take place by an inside-out evagination of the INM followed by approximation of the INM and ONM and subsequent fusion [118,119]. Although components of ESCRT-III have been implicated in this process, other cellular proteins have also been shown to have a role in NPC formation. Two of these, Torsin A and Torsin B, both members of the AAA+ ATPase superfamily of proteins, have been studied in the context of herpesvirus egress [70,120]. These proteins are localized to the PNS in interphase cells, and similar to ESCRT-III, the Torsins have been proposed to have a role in maintaining the nuclear envelope. Mutations in the gene encoding Torsin A, in both humans and in a transgenic mouse model of a human disease associated with loss of Torsin A function, led to altered NEC structure and blebbing of the INM [121,122]. A modest decrease in HSV replication in cells overexpressing Torsin A has been reported [70]. More recently, deletion of both Torsin A and Torsin B resulted in decreased PRV replication early in infection and, more interestingly, an accumulation of primary enveloped capsids in the PNS [120]. These enveloped particles were not associated with the ONM but appeared to remain attached to the INM [120]. These most recent findings are provocative in that retention of primary enveloped particles in the PNS suggested that Torsins could contribute to the transit through the PNS and de-envelopment at the ONM. 

Torsin A has also been shown to play an important role in the assembly of the LINC complexes, possibly through its interactions with the nesprins 1–3 and SUN proteins [120]. As described previously, the canonical LINC complex consists of a trimer of KASH domain proteins (nesprins), which are inserted in the ONM, and a trimer of SUN proteins that reside in the INM and act as a receptor for the C-terminal KASH domain of the nesprin (Figure 2) [26,30,123]. Recent studies show that the LINC complex forms 6:6 branched assemblies consisting of three KASH domain protein dimers and two SUN domain protein trimers [124]. Crosslinking of cytoskeletal elements by nesprins in branched assemblies could allow for more robust distribution of mechanical forces along the nuclear envelope and transduction of signals from the cytoplasm and external environment.

Virus yield in PRV-infected cells overexpressing dominant negative forms SUN1 and SUN2 was decreased, and perhaps more interestingly, primary enveloped capsids accumulated in the PNS and in the ER [71]. This latter observation provided evidence that the altered function of the LINC complex could limit nuclear egress of PRV and at least partially phenocopy the loss of Torsin A in PRV-infected cells [71,120]. These authors also reported that overexpression of these dominant negative forms of SUN1 and SUN2 led to a widening of the perinuclear space. The functions of Torsins in LINC complex dynamics, maintenance of the perinuclear space, and membrane fusion events during NPC assembly fusion in normal cells all suggest that these proteins could also have a role in herpesvirus transit through the PNS and ONM during nuclear egress. 

In the context of HCMV infection, Buchkovich et al. described a decrease in the steady state levels of the LINC proteins Sun1 and Sun2, which was correlated with an increase in the thickness of the PNS [4]. This finding is reminiscent of results from the knockdown of SUN protein expression in PRV-infected cells described above [71,120]. More recent studies have provided evidence of a potential function(s) of the LINC complex in the context of infection-induced nuclear rotation, a cellular response that has also been reported to occur during cell migration and secondary to mechanical stress [8,27,28]. These authors observed the accumulation of Sun1 at the nuclear membrane in close proximity to the cytoplasmic AC at late times post-infection, similar to the localization of condensed chromatin [8]. Interestingly, these authors previously reported that nuclear rotation in infected cells preceded the maturation of the cytoplasmic AC [16], a finding that could be interpreted as evidence that nuclear and cytoplasmic phases of HCMV assembly were coordinated through nuclear-cytoplasmic signals, a proposed function of the LINC complex [8]. With the exception of these observations on the potential contribution of the LINC complex in HCMV nuclear egress, there are few, if any, other published data available that have related the activity of cellular proteins to the transit of HCMV through the PNS and ONM. 

## 5. Summary

There has been considerable progress in defining the mechanism of egress of herpesviruses from the nucleus, including the role of individual virus-encoded proteins in egress. However, the contribution of cellular proteins to nuclear egress remains less well understood and an area of active investigation. Several cellular proteins and protein complexes have been shown to play a role in nuclear egress, and in some cases, specific function(s) in egress have been assigned. Moreover, the contribution of cellular proteins to egress in many cases appears redundant; thus, it has been difficult to establish robust phenotypes following conventional loss-of-function approaches used to study individual proteins. This redundancy in function can likely be accounted for by a similar redundancy in function of these cellular proteins in the homeostasis of uninfected interphase cells. In addition, efficient egress could also require combinatorial functions of several cellular proteins, thus further limiting conventional approaches commonly used for the definition of the function of a single cellular protein. In the case of HCMV, efficient nuclear egress is almost certainly dependent on the contribution of cellular responses to the infection if nuclear events during egress parallel the remarkable modifications of the host cell architecture, organelles, and intracellular trafficking pathways during HCMV replication. Defining the coordinated activities of host cell proteins and viral proteins during egress will provide an opportunity to extend current understanding of the role of cellular proteins in trafficking of macromolecules in and out of the nucleus.

## Figures and Tables

**Table 1:** Viral and cellular factors involved in nuclear egress.

	Role during Infection	Citation
*VIRAL NUCLEAR EGRESS COMPLEX*		
*pUL50/53*	Core NEC, forms hexameric ring structure that can deform membranes and perform membrane scission	[36,49,50,51,52,53,54]
*pUL93/pUL77*	Capsid vertex components 1/2, potential adaptor proteins binding to core NEC, required for encapsidation	[55,56]
*pUL97*	Conserved herpesvirus protein kinase, phosphorylates lamins and NEC components, possibly host proteins; disrupts lamin polymerization	[46,57,58,59]
*NEC-DIRECTED TRANSIT THROUGH INM*		
*Lamins*	Phosphorylated by viral and possibly cellular kinases; focal disruption of lamin network required for egress	[46,57,60,61,62,63,64]
*WDR5*	Required for efficient egress by unknown mechanism; may remodel chromatin and regulate nuclear architecture	[65]
*Protein kinase C*	Host kinase that phosphorylates lamina and disrupts lamin polymerization	[45,61]
*p32*	Recruited to INM by interactions with NEC; binds lamin B receptor; recruits UL97 to NEC	[62]
*Nuclear actin/Myosin Va*	Required for efficient transit of capsids to INM	[66,67]
*ESCRT-III*	Required for efficient egress in α-herpesviruses; repairs nuclear membrane ruptures	[21,23,68]
*p53*	Regulates expression of core NEC protein UL53	[47,48]
*Emerin*	Polarized expression in HCMV-infected cells; interacts with NEC; reduced expression disrupts AC formation	[8,35]
*TRANSIT FROM THE PNS THOUGH THE ONM*		
*Viral gB*	In α-herpesvirus infected cells, reported to be required for membrane fusion at ONM	[69]
*Torsin A*	ATPase; may regulate LINC complex; deletion causes accumulation of capsids in PNS in α-herpesvirus infected cells	[70]
*LINC COMPLEX*	Sun2 levels downregulated by infection; Sun1 localization polarized, levels may be downregulated; reduction in expression correlated with dilation of PNS; expression of Sun dominant-negative proteins reduces virus yield	[4,8,71]

## Data Availability

Not applicable.
